# Qualitative study of practices and challenges when making a diagnosis of asthma in primary care

**DOI:** 10.1038/s41533-019-0140-z

**Published:** 2019-07-17

**Authors:** Adeola Akindele, Luke Daines, Debbie Cavers, Hilary Pinnock, Aziz Sheikh

**Affiliations:** 1 Asthma UK Centre for Applied Research, Usher Institute of Population Health Sciences and Informatics, The University of Edinburgh, Edinburgh, UK; 2 Cancer and Primary Care Research Group, Usher Institute of Population Health Sciences and Informatics, The University of Edinburgh, Edinburgh, UK

**Keywords:** Diagnosis, Asthma

## Abstract

Misdiagnosis (over-diagnosis and under-diagnosis) of asthma is common. Under-diagnosis can lead to avoidable morbidity and mortality, while over-diagnosis exposes patients to unnecessary side effects of treatment(s) and results in unnecessary healthcare expenditure. We explored diagnostic approaches and challenges faced by general practitioners (GPs) and practice nurses when making a diagnosis of asthma. Fifteen healthcare professionals (10 GPs and 5 nurses) of both sexes, different ages and varying years of experience who worked in NHS Lothian, Scotland were interviewed using in-depth, semi-structured qualitative interviews. Transcripts were analysed using a thematic approach. Clinical judgement of the probability of asthma was fundamental in the diagnostic process. Participants used heuristic approaches to assess the clinical probability of asthma and then decide what tests to do, selecting peak expiratory flow measurements, spirometry and/or a trial of treatment as appropriate for each patient. Challenges in the diagnostic process included time pressures, the variable nature of asthma, overlapping clinical features of asthma with other conditions such as respiratory viral illnesses in children and chronic obstructive pulmonary disease (COPD) in adults. To improve diagnostic decision-making, participants suggested regular educational opportunities and better diagnostic tools. In the future, standardising the clinical assessment made by healthcare practitioners should be supported by improved access to diagnostic services for additional investigation(s) and clarification of diagnostic uncertainty.

## Introduction

Over-diagnosis and under-diagnosis of asthma are common.^1–4^ In Canada, asthma was ruled out in 33% of people with doctor-diagnosed asthma.^3^ Meanwhile, in the Netherlands 21% of people were underdiagnosed by their general practitioners (GPs).^4^ Consequences of under-diagnosis are a lack of treatment, with potentially avoidable morbidity. Over-diagnosis exposes patients to unnecessary side effects of treatment(s) and contributes to increased healthcare costs.^5^

In the United Kingdom (UK), asthma affects up to 5.4 million people, causes >1000 deaths annually and accounts for an estimated £1.1 billion in annual National Health Service (NHS) spending.^6,7^ As most asthma diagnoses are made in primary care, it is particularly important to explore the misdiagnosis of asthma in this setting.

Asthma diagnosis is difficult for several reasons. First, asthma is a variable condition that relapses and remits, so symptoms and signs are often not present during a routine healthcare appointment.^8^ Second, there is no gold-standard diagnostic test for asthma.^9^ In practice, peak expiratory flow measurements and spirometry are used to demonstrate features of asthma, such as variable airway obstruction and reversibility of airway obstruction with bronchodilator drugs.^10^ However, these tests cannot always exclude asthma as results may be normal when patients are asymptomatic.^8,10^ Challenge tests with histamine or methacholine have greater sensitivity but are often not tolerated well by patients and are not undertaken in community settings.^11,12^ Third, guideline recommendations are not always compatible with working systems in primary care. For instance, objective testing is not always available in the community, and even if available, testing cannot always be done due to time pressures.^10^ This is particularly relevant in the UK, given that the recent National Institute for Health and Care Excellence (NICE) guidelines recommend fractional exhaled nitric oxide (FeNO) and spirometry as core tests for making a diagnosis in all patients.^9^ FeNO indicates eosinophilic inflammation and raised FeNO levels support a diagnosis of asthma.^13^ However, FeNO levels may also be elevated in males, people with allergic rhinitis, rhinovirus infection and following intake of nitrate-rich foods such as green vegetables, which can lead to false positive results. In addition, FeNO levels may be reduced in smokers and in people who have had steroid treatment raising the possibility of false negatives. Furthermore, it is currently unclear how best to implement FeNO in routine UK primary care settings.^14^

Most previous studies on misdiagnosis of asthma have used a quantitative approach to demonstrate the proportion of people who are over- or under-diagnosed^1–4^ but have not sought to understand why these misdiagnoses occur. Qualitative studies enable exploration of the factors influencing diagnostic decision-making and the challenges involved.^15^ Therefore, the aim of this study was to explore the experiences and perspectives of doctors and nurses when making a diagnosis of asthma in primary care. Specifically, our objectives were to:

- Determine the diagnostic process used by each clinician and understand which elements from a patient’s history, tests and clinical record are most valued.
- Identify the challenges faced by clinicians when making a diagnosis.
- Explore sources of asthma misdiagnosis and ideas to help improve the accuracy of diagnosis of asthma in primary care, including the use of clinical prediction tools.

## Results

Ten GPs and five practice nurses took part in face-to-face interviews between February and April 2018. (Tables 1 and 2). Interviews lasted between 25 and 40 min. Five major themes were generated from the thematic analysis: approach to diagnosis, factors affecting the approach to diagnosis, challenges affecting diagnoses, misdiagnosis of asthma, and suggestions for and opinions on potential future approaches to asthma diagnosis.

**Table 1:** Participant characteristics

Characteristics	Participants (*n* = 15)
Sex	
Female	11
Male	4
Role	
GP	10
Nurse	5
Age (years)	
<40	8
41–50	3
51–60	3
>60	1
Role in practice	
Partner	2
Salaried	5
Locum	2
Out-of-hours	1
Practice nurse	4
Nurse practitioner	1
Interest in asthma	
Diploma in asthma	5 (all nurses)
Asthma lead	1 (GP partner)
Years since qualification, median (range)	13 (0.5–37)

**Table 2:** Practice characteristics

Practices (*n* = 10)	*n*
Practice list size^a^	
<5000	1
5000–10,000	7
>10 000	2
Practice Index of multiple deprivation^b^	
1–5 (more deprived)	8
6–10 (less deprived)	2

^a^ISD Scotland: https://www.isdscotland.org/Health-Topics/General-Practice/Workforce-and-Practice-Populations/_docs/Practice_ContactDetails_Jan2018_final.xlsx?14:33:27

^b^Scottish Government: http://www.gov.scot/Resource/0050/00505244.xlsx

### The diagnostic process

Participants used the probability of asthma determined from the clinical history to guide their diagnostic approach. Patients with a high probability of asthma were those with wheeze, family history of asthma, personal or family history of atopy, nocturnal symptoms and/or exertional symptoms.

So the things that would make me think “yes, this is more likely to be asthma” is themselves having had a history of being a viral wheezer as a child, having atopy in the family, having eczema in the family or themselves, allergies to pets and things. And a typical history of asthma, as in worsening with certain things, worsening with eczema, worsening with cold air, for example. Not just wheezy all the time. GP 7

When patients’ symptoms suggested a high probability of asthma, most participants carried out further, immediately available, testing e.g. with a peak flow meter.

If I think it’s a very convincing clinical picture […]. Then if I think their spot peak flow is reduced, for example, and there’s no intercurrent infection to explain it, if I [think it is] high probability I would be fairly confident about just empirically starting some treatment and then monitoring … essentially a trial of treatment. GP 6

The tests available to participants in their practices were peak flow meters and spirometry. Most participants used peak flow meters as the first line of investigation though factors including age and compliance influenced which test was used.

In younger children, participants recognised that objective testing was difficult, though sometimes used peak flow measurements to arrive at a diagnosis.

If they’re able to, kids-wise, I’d probably go down the serial peak flow route before I would think about spirometry. […] Because really, like I say, even trying to get them to do a proper peak flow can be difficult. Nurse 2

Meanwhile in adults, GPs had greater confidence to request spirometry compared to nurses, if there was diagnostic uncertainty or if patients were unlikely to be compliant with peak flow measurements.

So if it’s someone that can do a simple peak flow meter, I would do that in the first instance. […] Whereas if they’re probably not compliant or they’re too young or you’re worried that there might be… they’re smokers, you know, it’s not kind of classic asthma symptoms, then usually they’re referred to spirometry. GP 10.

Six out of the ten practices involved in this study had access to in-house spirometry. However, some GPs preferred hospital testing to in-house spirometry because they were more confident with the results and they had concerns about adding to workload pressures for their staff.

We do have spirometry machines here but we’d still tend to get them done at the hospital. […] we would trust the results more. And also time; our nurses are fully booked […] the more things like that we do here, it just becomes unmanageable. Like we don’t have enough nurses’ slots for dressings or other stuff. GP 5

Participants used a trial of treatment as a diagnostic approach at different stages of the diagnostic process. Some participants used the trial of treatment to confirm the diagnosis when spirometry and peak flow measurements were normal but the clinical probability of asthma was high.

Yeah, so if I felt they were pointing more towards asthma but their spirometry was still normal, and say their chest X-ray was normal as well, then I would consider a trial of treatment. GP 8

Meanwhile, some participants used a trial of treatment without objective testing as the main diagnostic tool, if the clinical probability of asthma was high. They explained that this approach was based on current British Thoracic Society/Scottish Intercollegiate Guidelines Network (BTS/SIGN) guideline recommendations.^16^

Certainly the diagnosis part has all changed because we can now look at the probability and go directly into trial of treatment rather than saying “right, well, we’ll do peak flow diary on everyone”. Nurse 1

None of the participants in the study had FeNO available in their practice. Participants understood the potential benefits of FeNO testing, though most were worried about the cost of introducing FeNO into primary care.

I do feel it would be a step forward for us being more proactive and positive with tests if it was available but I suppose it’s finances to supply the FeNO. Nurse 4

Chest X-ray and full blood count including eosinophils were other non-routine tests used by participants to confirm or refute asthma.

### Challenges of asthma diagnosis

Participants felt that diagnosis was difficult if patients were asymptomatic at the time of testing, as objective tests were often normal.

So quite often if they’re not symptomatic when they come here so you’re not going to get any change in reversibility. So it’s not always as straightforward as that – sometimes it is but sometimes it’s not. Nurse 4

Nurses also felt that having asymptomatic intervals made it challenging to arrive at a final diagnosis, as patients often failed to come for follow-up appointments.

Sometimes you’ll send them away with serial peak flow monitors to do readings and they don’t always appear back. Because I think maybe sometimes if you’re looking at their symptoms, maybe their symptoms have improved and they’ve not bothered doing it and they just don’t bother coming back. Nurse 2

Participants found it difficult to differentiate between asthma and other closely related conditions, especially at the extremes of age. In children, participants found it difficult to differentiate between multiple presentations of viral-induced wheeze and asthma.

Viral-induced wheeze in asthma [is difficult]. Because you could genuinely have, I think, a little bit of wheeze with a virus that’s not asthma and you can genuinely have asthma that is made worse by a virus. GP 9

At the other extreme, participants were especially worried that many other conditions could cause symptoms in older people.

I mean, when you get older, anything can cause a shortness of breath on exertion, can’t it? It could be cardiac, anaemia or kind of respiratory as well. They’re much more likely to have COPD if they’ve been smokers. Or you can get kind of COPD and asthma kind of overlap as well. GP 2

Furthermore, in adults, participants sometimes found it difficult to differentiate exercise-induced asthma from a normal response to exercise.

One of the things I find difficult is exercise-induced. You know, you get a lot of people saying, “oh, I get short-of-breath when I exercise” and when you take their history it’s very much like “when I’m working hard I get short-of-breath” and you think “well, that’s just exercise, that’s what happens when you run up a hill, you get short-of-breath, you know.” GP 7

Some participants felt that arriving at an asthma diagnosis was difficult to achieve with the time pressures in general practice.

Yeah, in a single standard appointment, which is usually 10 min, it’s fairly difficult to make a definitive diagnosis. So that’s often why I use tools and techniques, such as the peak flow diary or a trial of treatment. GP 6

### Understanding misdiagnosis

Under-diagnosis was perceived to be less of a problem than over-diagnosis. Participants felt that if people did not present, they could not make a diagnosis.

I mean, there could be plenty of people out there who never see their GP who might have a bit of asthma that, you know, GPs would never know about because they never come and they’d never report the fact they’d got exertional symptoms. GP 2

Participants explained that, because salbutamol provides symptomatic relief, trials of salbutamol inhalers were commonly given to patients with respiratory symptoms pending a definite diagnosis.

One reason for participants continuing to prescribe treatment without a diagnosis was the fear of incorrectly labelling a patient with asthma.

No [I don’t find diagnosis easy], I mean, I hesitate to put on a repeat prescription for salbutamol and I might even do it without putting the ‘asthma’ word on it. GP3

Some participants expressed concern that one-off prescriptions of salbutamol may become repeat prescriptions based on patient request if there were no clear records of the basis of the initial prescription.

So they have an acute prescription for salbutamol and then maybe they go to another GP and say “well, I have to keep on getting my acute, can you put it on repeat?” And then it almost seems like the next GP that sees them they’ll start treating them as though they do have asthma. GP 1

Although some participants found asthma diagnoses time consuming, other participants expressed concern that misdiagnosis may occur if a diagnosis was made too quickly.

And looking back, when I’m saying people aren’t asthmatic anymore and saying ‘asthma resolved’, it’s that the doctor just seems to have come to a quick decision. GP 5

This GP and some other participants described ‘using time as a diagnostic tool’.

### A future approach to asthma diagnosis

Participants suggested regular educational updates as a way of improving the diagnostic accuracy of asthma in general practice.

I think GP education’s very important. […] I think having regular CPD (Continuous Professional Development) about that’s really important, because we get de-skilled very quickly, and just seeing what’s there. GP 10

Other suggestions to improve the diagnosis of asthma in primary care included having a clearer diagnostic process and more tests.

I think the more tests we can have, the better. I think patient education and obviously healthcare education, because it isn’t straightforward. Obviously, they’re tools we can all use but the more tools, the better, I feel. Nurse 4

I mean, it’s all about if there was a clear diagnostic process which is standardised and put in everyone’s face and clearly set out then that would be helpful. GP 4

Interestingly, one nurse suggested the use of a tool to help her quantify the risk of asthma even before being asked about clinical prediction tools.

Maybe if there was a kind of tool that helped bring all these different factors… you know, like when we’re looking at symptoms and we’re looking at their spirometries and all that, […], just to kind of look at it all and help us think “oh, that IS a high probability” or there is a kind of suspicion of asthma but we’re not sure, that kind of thing. Nurse 2

### Proposed clinical prediction tool

Participants were specifically asked to comment on the use of clinical prediction tool and diagnostic hubs to improve asthma diagnosis. All the nurses interviewed liked the idea of an additional tool that guided their decision-making process.

Yeah. I mean, certainly, absolutely, it can take you, I suppose, quite easily into the high probability section and then from there you would know what to do. But obviously it’s a clinical thing, isn’t it, as well? But no, absolutely, I think any tool that helps you decide that there’s… because there can be a sort of ambiguity. Nurse 1

By comparison, most GPs preferred using their clinical judgement to diagnose asthma and were reluctant to use a tool that provided the probability of asthma.

I think at the moment it’s very much gut feeling and using time as a diagnostician and asking the patient about their symptoms. And because it’s history-based, it’s very much kind of you’re asking the patient preferences and the patient’s got more autonomy. […] And I think introducing calculators makes general practice a lot less sensitive, gut feeling, what the patient wants and much more protocol-driven… I think with asthma specifically, because it’s so history-based, there’s so much on gut feeling, they’re probably not a good thing. GP 2

There were, however, some GPs who were open to using a prediction tool if it was validated to have a high positive predictive value, promoted by guidelines, easy to use and self-populated with available data from the electronic patient record system used by GPs.

I mean, all of these tools, if they’re easy to use and simple, can help guide our clinical judgement. Absolutely, yeah, why not? So they have to be easily-accessible […]. I suppose the other point is, you wouldn’t use it if it’s not validated and no good so it has to have a high usefulness. It can be as easy as anything but if it’s not very sensitive then there’s no point. GP 7

### Proposed diagnostic hubs

Participants were open to using diagnostic hubs as a centre for diagnostic queries. They thought it would be helpful to provide continuity of testing as they envisioned that the same person might do the history and investigations.

But I can see where being in a central place for… you would get the continuity of all the testing but it would have to be done over a day, they would have to go back and get the results and show them, Yeah. Nurse 1

The advantage of diagnostic hubs to provide easy access and reduce waiting times was also mentioned.

It would definitely be useful in the ease of accessing resources like that or the provision in terms of location and accessibility and waiting times. If that’s easier then those services will get used more and they may well be helpful. GP 6

However, some participants were worried about the deskilling of primary care practitioners if all patients with suspected asthma were sent to a hub for diagnosis.

I suppose for us it might de-skill us slightly because then we wouldn’t be seeing these patients very regularly and then you wouldn’t be using… I suppose you’d still be doing your spirometry and all these things for your COPDs but aye (yes), it would maybe de-skill us slightly. Nurse 2

A GP suggested that sending patients to a diagnostic hub to confirm asthma would reduce GPs’ confidence in the ability to diagnose asthma.

Yeah, it does really de-skill. [..] Or actually, maybe most of it would be lack of confidence, really. But yeah, I think kind of getting a diagnosis in a hub would definitely de-skill. And I think asthma should be kind of bread-and-butter for GPs. GP 2

## Discussion

We aimed to explore the experiences and perspectives of doctors and nurses when making a diagnosis of asthma in primary care. Participants used heuristics to assess the clinical probability of asthma to decide if and which of peak flow measurements, spirometry and trial of treatment would be appropriate for each patient. Participants found asthma diagnosis difficult at the extremes of age; when features of asthma overlapped with other conditions such as viral illnesses and chronic obstructive pulmonary disease (COPD), especially in those too young for tests. Under-diagnosis was due to patients’ decision to present (or not); meanwhile, having patients on treatment without a diagnosis contributed to over-diagnosis. Participants suggested that regular education and better diagnostic tools would improve asthma diagnosis in primary care.

Most participants found the use of tests to refine the probability of asthma helpful. However, some participants felt that if the probability of asthma was high, treatment could be started without objective testing. This approach resonates with the BTS/SIGN guidelines, which recommends determining the initial probability of asthma using a structured clinical assessment.^16^ If the probability of asthma is high, a carefully monitored initiation of treatment can be considered, using objective measurements of lung function and/or a validated control questionnaire to confirm (or refute) the already high probability of asthma.^16^ However, there is no evidence-based definition of ‘high probability’ leaving clinicians with a lack of clear guidance on the predictive value of common symptoms and items in the clinical history, the threshold at which further tests are needed and the order in which investigations should be performed. In order to reduce misdiagnosis, further research is needed to quantify probabilities and develop tests that provide more accurate results and take little time or resources to perform.^17^

The Bayesian terminology of probabilities reflects the recognised clinical strategy for making a diagnosis.^18^ Most of the GP participants were comfortable with this approach and felt uncertain about whether clinical prediction tools would be beneficial. In comparison, other participants, including all the nurses interviewed, were open to the advantages of a system that guided their clinical judgement as long as it was ‘easy to use’. To be acceptable to clinicians and adopted in routine practice, clinical prediction tools will not only have to offer practical advice about (available) tests and deliver an accurate probability of a diagnosis but will also be more acceptable if they operate within current diagnostic paradigms.^19^

Diagnostic overlap sometimes made asthma diagnosis difficult for participants in this study. In children, this overlap was between viral-induced wheeze and asthma. Meanwhile, in adults, participants worried about conditions such as COPD, echoing widely expressed views.^20–22^ Contemporary understanding of ‘the asthmas’ is moving towards considering asthma as an initial description, which is then refined into phenotypes with different disease trajectories, and underlying airway inflammation.^23,24^ The future of asthma diagnosis (at least in resource-rich settings) may therefore move away from the label of ‘asthma’ to delineating phenotypes or ‘treatable traits’ requiring additional tests (such as FeNO). However, none of the participants in this study had FeNO available within their practice and several participants expressed concern over the resource implications of providing FeNO. Indeed, despite having access to spirometry, some participants referred patients to secondary care for spirometry for reasons of resource as well as quality.

Diagnostic hubs are a model of care that offer an alternative to provision of respiratory investigations within the practice potentially offering not only expertise but also enabling timely introduction of new approaches to diagnosis and phenotyping.^25^ Evidence for these approaches will need to include clinical and cost-effectiveness considerations to guide implementation.

Fear of incorrectly labelling patients with asthma may explain under-diagnosis of asthma for some patients; several GPs adopted symptomatic treatment rather than diagnostic labels due to lack of certainty.^26,27^ Although avoiding labels is not unique to asthma,^27^ the implications of symptomatic relief instead of a diagnosis may delay provision of effective interventions, such as supported self-management.^28^ In contrast, difficulty communicating uncertainty to patients may lead some clinicians to attach a label before a diagnosis is confirmed thereby risking over-diagnosis. Some participants suggested that there was a risk that a ‘suspected asthma’ code could become an ‘asthma’ code by default if patients’ requests for repeat prescriptions were not scrutinised carefully—a risk potentially exacerbated by a lack of continuity of care. This emphasises the importance of guideline recommendation to ‘record the basis on which the diagnosis was made’.^16,29^

By using a qualitative approach, this study has provided insights into primary care professionals’ views, experiences, frustrations and ideas for improvement when diagnosing asthma. The information gathered can inform healthcare providers and policymakers, enabling them to recognise and address practical challenges. Our participants represented a range of experience and practice demography, but all were within NHS Lothian (and thus had access to secondary/tertiary diagnostic services); GPs working in rural or remote areas may have other perspectives. Our findings may be applicable to other similar healthcare systems, though globally, approaches to diagnosis will vary.^30,31^

The influence of the interviewer in qualitative studies must be considered. A.A. conducted the interviews as a medical student. This allowed her to ask more ‘why’ questions about decision-making than an experienced clinician may have omitted if they felt the answer was obvious. Nevertheless, some participants assumed that questions were testing their knowledge. To deal with this, questions were phrased to emphasise experience, yet some responses might have been provided to match expectations rather than a true reflection of day-to-day practice. In qualitative studies, sample size may be considered adequate when it is unlikely that further interviews will yield new responses.^32^ For resource reasons, our study was limited to obtaining the views of 15 primary care practitioners. This was sufficient to reach data saturation with respect to the current diagnostic processes used, but we may not have heard all views on misdiagnosis and new approaches to asthma diagnosis.

Policy and health service concerns about misdiagnosis are reflected in the perceptions of individual healthcare professionals who perceive the diagnosis of asthma to be challenging and (in the absence of a diagnostic test) rely on clinical assessment of probabilities. The lack of evidence-based algorithms for achieving a robust diagnosis, and the evolution of the concept of ‘treatable traits’, have added to diagnostic uncertainty, which may contribute to under- and over-diagnosis. Decision support systems that aid clinicians to assess probabilities more accurately and optimise the use of existing diagnostic tests may be an effective approach to reducing misdiagnosis. The observation that practice nurses were more inclined towards a tool that guided their clinical judgement than GPs should be considered in the design of future diagnostic aids. Other recommendations to improve the accuracy of asthma diagnosis include greater provision of professional education and improving access to investigations such as spirometry, potentially through diagnostic hubs.

This study describes the current approach of determining the probability of asthma based on a clinical assessment corroborated by peak expiratory flow measurements, spirometry and/or a trial of treatment. Challenges related to time and resource pressures in general practice and the overlapping features of asthma with other conditions. Future models of care need to provide cost-effective access to accurate (potentially novel) tests, and institute diagnostic services that guide clinicians through evidence-based algorithms and that reflect our evolving understanding of asthma and its phenotypes are needed.

## Methods

### Design

This is a qualitative study using semi-structured interviews with UK GPs and practice nurses in Lothian, Scotland.

### Ethics

The study was reviewed by the Research and Development Committee, Queen’s Medical Research Institute Edinburgh who approved the study and considered that no ethical review was required for interviews with health professionals. (Lothian project R&D no: 2017/0292)

### Participant recruitment

GPs and practice nurses working in NHS Lothian, Scotland were invited to take part in the study through local primary care networks and word of mouth. From this group of potential participants, we selected a diverse sub-sample; a mix of professionals based on gender, years of experience and clinical role. Interested GPs and practice nurses were emailed the information leaflet explaining the reasons for the study and potential uses of the results obtained from the study. All participants provided written informed consent.

### Data generation

A topic guide, with prompts informed by existing literature, was used to answer the research questions (Supplementary Table 1). Interviews were carried out by A.A., a postgraduate medical student at The University of Edinburgh with close support and training from L.D. and A.S. (both clinical academics). In preparation for data collection, A.A. completed three pilot interviews with GP colleagues, receiving feedback from the interviewee and the research team. Regular team meetings were held throughout the study period to review transcripts and refine interview technique.

GPs and practice nurses were asked how they made a diagnosis of asthma, the investigations available and the challenges they faced when making a diagnosis of asthma. In addition, participants were asked about their perspectives on asthma misdiagnosis and their suggestions for improving the diagnosis of asthma in primary care. Participants were also asked to comment specifically on ideas that have been suggested to improve asthma diagnosis including a clinical prediction tool to determine the probability of asthma during consultations and provision of diagnostic hubs that serve as a ‘one-stop shop’ for patients to receive all necessary assessments.^9,25^

The interviews took place in clinics and university meeting rooms. All interviews were digitally audio-recorded and transcribed verbatim. There were no repeat interviews. Field notes made after each interview were also used to record relevant contextual issues, such as rapport and questions raised.

### Data analysis

Transcripts were analysed using a thematic approach as described by Braun and Clarke.^33^ Initially, each transcript was studied, and sections of text were coded. In the early stages of analysis, A.A. in discussion with L.D., A.S. and D.C. drew up a provisional coding framework guided by the research questions. Codes were then aggregated into major themes and subthemes using NVivo 11 (QSR international). Following regular discussion with the research team, the coding framework was continuously refined to include new themes and subthemes identified as the transcripts were reread in order to reflect the entire data set (see Supplementary Table 2 for final coding framework). The consolidated criteria for reporting qualitative research (COREQ) was used to guide reporting (Supplementary Table 3).

### Reporting summary

Further information on experimental design is available in the Nature Research Reporting Summary linked to this article.

## Supplementary information

Supplementary Information

Reporting Summary

## Data Availability

We do not have consent to share interview data. Further information may be available from the corresponding author.

## Supplementary information

**Supplementary information** accompanies the paper on the *npj Primary Care Respiratory Medicine* website (10.1038/s41533-019-0140-z).
