# Supplementary material for: Qualitative study of practices and challenges when making a diagnosis of asthma in primary care
Source: NPJ Prim Care Respir Med. 2019 Jul 17;29:27. doi: 10.1038/s41533-019-0140-z (PMC6637121; doi:10.1038/s41533-019-0140-z)
Supplement: Supplementary file 1 — Supplementary Information [file 41533_2019_140_MOESM1_ESM.pdf]

Supplementary information

Qualitative study of practices and challenges when making a diagnosis of asthma in primary care

Supplementary Table 1: Topic guide

Opening question	Prompts
How long have you worked at the practice?	
Can you tell me how you diagnose patients in asthma?<br>Can you tell me about a time you have diagnosed asthma?<br>Are you confident to make a diagnosis of asthma?<br>What about the case stood out for you?<br>(Nurses: Would you be comfortable making a diagnosis of asthma?<br>What do you think would be important for making a diagnosis?)<br>Can you think of another scenario where the patient/process was quite different?<br>Why do use that strategy?<br>How has your strategy changed over the years?<br>How have you come to use this approach?<br>What has influenced this approach over the years?	• Explain strategy<br>• History<br>• Exam (wheeze on auscultation)<br>• Do you classify patients according to probability of asthma (high, intermediate, low)?<br>• Do you classify patients into types of asthma a (topic, late-onset, non-allergic, exercise induced, cough variant, obesity related, occupational)<br>• Children<br>• What has influenced your approach? (Guidelines, experience, learnt from colleague)<br>• Have you refined your strategy over the years (what changes, why did you make those changes)<br>• If not (haven't refined), why?<br>• What was the role of Quality Outcomes Framework (QOF) in diagnosing asthma, if any?<br>• No strategy-how do you make diagnosis?
**Verify or explore differentials**	
In a situation where you have a patient who is likely to have asthma, what do you do next?<br>What tests are available to you?<br>To confirm?<br>If tests/trial of treatment are normal?	• Tests (which tests- Peak flow, spirometry, challenge tests, FeNO)<br>• Tests availability (where are the tests done?)<br>• Do you use spirometry? Do you always use spirometry?<br>• Spirometry- how do you access spirometry, what is the form of results, Do you always use reversibility tests, Who interprets the test, if nurse practitioner does it, is he/she trained?<br>• What do you do if spirometry is normal but there is a high clinical suspicion?<br>• Trial of treatment-do you use a trial of treatment. What would the trial of treatment be? When do you review after a trial of treatment?<br>• Review diagnosis - after how long?<br>• I have been hearing that QOF made GPs and nurse do more spirometry. What do you think will happen now that QOF is no longer in place?

Opening question	Prompts
**After initial work up (suspected / confirmed diagnosis)**	
Is there anything you do after a diagnosis has been confirmed?	• Code: Description of what evidence diagnosis is based on, suspected asthma<br>• The review process
What challenges are there in making an accurate and timely diagnosis of asthma?	• Appointments-10 minute time limit<br>• Atypical subtypes/presentations: obese patients, patients with GORD, post viral cough<br>• Conflicting guidelines<br>• Availability of tests<br>• Challenges with reviewing patients-How do you code patients that get better with trial treatment but don't come back?<br>• Referral to secondary care/relationship with secondary care<br>• Reviewing or questioning existing diagnoses<br>• How do you tackle these challenges?
**Misdiagnosis generally**	
Are you aware of any issues around misdiagnosis of asthma?	• Under diagnosis<br>• Over diagnosis
What do you think would help to reduce issues around misdiagnosis of asthma in the future?<br>Is there anything you do in your practice that you think should be widely available?	• Diagnostic support services? centralised hub to capture all necessary tests needed for a diagnosis<br>• What are your thoughts on sending patients to a community based respiratory assessment unit as opposed to the hospital for spirometry when you are unsure of the diagnosis?<br>• Would you be happy to send patients even if you were sure of the diagnosis?<br>• Mobile technology-e.g. recording patients<br>• New tests: FeNO<br>• What do you think of a software that will help to diagnose asthma<br>• Decision support tools: Clinical prediction calculator e.g. ASSIGN score that predicts people likely to develop heart disease in the next 10 years<br>• What features of a clinical prediction rule make you likely to use it?
**Any other ideas about improving asthma diagnosis?**	

Supplementary Table 2: Coding framework

Name	Sources	References
**Approach to diagnosis**	0	0
Confidence about making diagnosis	7	15
Probability of asthma	12	29
Clinical history	14	28
Examination	10	12
Investigations	0	0
Spirometry	15	53
Peak flow	14	42
Other	7	9
Trial of treatment	15	53
Referral to secondary care	15	36
Asthma phenotypes	6	6
Asthma codes	4	5
**Factors influencing the diagnostic process**	0	0
Patient population	0	0
Children	14	29
Old enough or elderly	9	12
Guidelines	11	24
Knowledge or Clinical experience	5	7
QOF	7	10
Smoking status	6	6
Patient preference	2	2
Organisation of healthcare	0	0
Training or updates	7	9
Role in practice	6	11
Compliance to do test	2	4
Professional's preference	1	1
**Challenges in the diagnostic process**	0	0
Guidelines	5	9
Time	8	16
Failure to attend appointments	6	9
Patient age group - children	9	15
Patient age - older patients	4	5
Variable nature of asthma	9	13
New patients with existing diagnoses	1	1
Lack of gold standard test	6	7
overlap with COPD and other diagnoses	9	16
Possible mild or exercise induced asthma	4	5
Fear of diagnostic labelling	3	4
Clinical variety of asthma	2	2
**Misdiagnosis**	0	0
Perceptions on misdiagnosis of asthma	14	25
Sources of overdiagnosis	11	28
Sources of underdiagnoses	4	4
Inhalers without diagnosis	5	11
Revisiting diagnosis	10	17
Suboptimal coding	2	3
**Opinions on future approach to asthma diagnosis**	4	6
Clinical prediction calculator	15	23
Diagnostic hubs	15	25
Opinions on FeNO	12	23
Asthma as a priority	3	5
Revisit asthma diagnoses	2	2
Education	2	2
Definitive diagnostic process	4	5
Use objective tools	2	2

Supplementary Table 3: COREQ (COnsolidated criteria for REporting Qualitative research) Checklist

Topic	Item No.	Guide Questions/Description	Reported on Page No.
**Domain 1: Research team and reflexivity**			
Personal characteristics			
Interviewer/facilitator	1	Which author/s conducted the interview or focus group?	19
Credentials	2	What were the researcher's credentials? E.g. PhD, MD	19
Occupation	3	What was their occupation at the time of the study?	19
Gender	4	Was the researcher male or female?	17
Experience and training	5	What experience or training did the researcher have?	19
Relationship with participants			
Relationship established	6	Was a relationship established prior to study commencement?	19
Participant knowledge of the interviewer	7	What did the participants know about the researcher? e.g. personal goals, reasons for doing the research	19
Interviewer characteristics	8	What characteristics were reported about the interviewer/facilitator? e.g. Bias, assumptions, reasons and interests in the research topic	19
**Domain 2: Study design**			
Theoretical framework			
Methodological orientation and Theory	9	What methodological orientation was stated to underpin the study? e.g. grounded theory, discourse analysis, ethnography, phenomenology, content analysis	20
Participant selection			
Sampling	10	How were participants selected? e.g. purposive, convenience, consecutive, snowball	19
Method of approach	11	How were participants approached? e.g. face-to-face, telephone, mail, Email	19
Sample size	12	How many participants were in the study?	5
Non-participation	13	How many people refused to participate or dropped out? Reasons?	19
Setting			
Setting of data collection	14	Where was the data collected? e.g. home, clinic, workplace	20
Presence of non-participants	15	Was anyone else present besides the participants and researchers?	20
Description of sample	16	What are the important characteristics of the sample? e.g. demographic data, date	5, 19, Tables 1&2
Data collection			
Interview guide	17	Were questions, prompts, guides provided by the authors? Was it pilot tested?	19, Supplementary Table 1
Repeat interviews	18	Were repeat inter views carried out? If yes, how many?	20
Audio/visual recording	19	Did the research use audio or visual recording to collect the data?	20
Field notes	20	Were field notes made during and/or after the interview or focus group?	20
Duration	21	What was the duration of the interviews or focus group?	5
Data saturation	22	Was data saturation discussed?	18
Transcripts returned	23	Were transcripts returned to participants for comment and/or correction?	
**Domain 3: analysis and findings**			
Data analysis			
Number of data coders	24	How many data coders coded the data?	20
Description of the coding tree	25	Did authors provide a description of the coding tree?	5
Derivation of themes	26	Were themes identified in advance or derived from the data?	20
Software	27	What software, if applicable, was used to manage the data?	20
Participant checking	28	Did participants provide feedback on the findings?	
Quotations presented	29	Were participant quotations presented to illustrate the themes/findings? Was each quotation identified? e.g. participant number	5-14
Data and findings consistent	30	Was there consistency between the data presented and the findings?	

Topic	Item No.	Guide Questions/Description	Reported on Page No.
Clarity of major themes	31	Were major themes clearly presented in the findings?	5
Clarity of minor themes	32	Is there a description of diverse cases or discussion of minor themes?	5-14

Developed from: Tong A, Sainsbury P, Craig J. Consolidated criteria for reporting qualitative research (COREQ): a 32-item checklist for interviews and focus groups. *International Journal for Quality in Health Care*. 2007. Volume 19, Number 6: pp. 349 – 357
